# 3-Sulfogalactosyl-dependent adhesion of *Escherichia coli* HS multivalent adhesion molecule is attenuated by sulfatase activity

**DOI:** 10.1074/jbc.M117.817908

**Published:** 2017-10-05

**Authors:** Fitua Al-Saedi, Diana Pereira Vaz, Daniel H. Stones, Anne Marie Krachler

**Affiliations:** From the ‡ Institute of Microbiology and Infection, School of Biosciences, University of Birmingham, Edgbaston B15 2TT Birmingham, United Kingdom and; the § Department of Microbiology and Molecular Genetics, University of Texas McGovern Medical School at Houston, Houston, Texas 77030

**Keywords:** adhesin, intestinal epithelium, mucin, mucus, protein-lipid interaction, Multivalent Adhesion Molecule, commensal, foraging, mammalian cell entry domain

## Abstract

Bacterial adhesion to host receptors is an early and essential step in bacterial colonization, and the nature of adhesin–receptor interactions determines bacterial localization and thus the outcome of these interactions. Here, we determined the host receptors for the multivalent adhesion molecule (MAM) from the gut commensal *Escherichia coli* HS (MAM^HS^), which contains an array of seven mammalian cell entry domains. The MAM^HS^ adhesin interacted with a range of host receptors, through recognition of a shared 3-*O*-sulfogalactosyl moiety. This functional group is also found in mucin, a component of the intestinal mucus layer and thus one of the prime adherence targets for commensal *E. coli*. Mucin gels impeded the motility of *E. coli* by acting as a physical barrier, and the barrier effect was enhanced by specific interactions between mucin and MAM^HS^ in a sulfation-dependent manner. Desulfation of mucin by pure sulfatase or the sulfatase-producing commensal *Bacteroides thetaiotaomicron* decreased binding of *E. coli* to mucin and increased the attachment of bacteria to the epithelial surface via interactions with surface-localized sulfated lipid and protein receptors. Together, our results demonstrate that the *E. coli* adhesin MAM^HS^ facilitates retention of a gut commensal by attachment to mucin. They further suggest that the amount of sulfatase secreted by mucin-foraging bacteria such as *B. thetaiotaomicron*, inhabiting the same niche, may affect the capacity of the mucus barrier to retain commensal *E. coli*.

## Introduction

The gastrointestinal tract is covered by a mucus layer, which forms a semi-diffusive barrier protecting the underlying epithelium against microbial and chemical insults. The mucus layer consists of two parts, a cell-attached and a loose mucus layer (1), which is the habitat of the commensal microbiota (2). Both are mainly made up of the mucin Muc2, a large gel-forming glycoprotein that is modified with *O-*glycans, although the levels and pattern of glycosylation vary with localization (3, 4). Muc2 is initially anchored to the intestinal cell surface and is shed into the lumen upon cleavage (5). The epithelium lining the intestinal tract is supported by a basement membrane containing extracellular matrix proteins, including fibronectin, collagen, and laminin, which is only exposed if the epithelium is breached by bacterial insult or mechanical disruption. In healthy individuals, bacteria are prevented from accessing the inner mucus layer and epithelial surface and are maintained at the luminal side of the mucus barrier (6). In contrast, the distal mucus layer, which undergoes fast renewal and turnover to maintain gut homeostasis (7), constitutes a distinct intestinal niche cohabited both by bacterial species that forage on mucus-derived glycans, such as *Bacteroides* spp. and species that lack mucolytic abilities, including commensal *Escherichia coli* (8, 9). The composition and activity of the gut microbiota, as well as functional competition in this habitat, can shape the mucus barrier in a way that determines microbe–host interactions (8–10). Aberrant production and *O-*glycosylation of Muc2 have been associated with increased bacterial penetration and thus elevated inflammatory responses both in rodent models and patients (11–13). The composition and localization of the gut microbiome are shifted in patients with inflammatory bowel diseases, and an overabundance of mucus-foraging species as well as a closer association of commensals, including non-mucolytic *E. coli,* have been reported (14). Although a causality between these two observations has not been firmly established, it has been hypothesized that the enhanced production of mucinolytic enzymes leads to compromised barrier integrity, which facilitates the relocation of microbes in close proximity of the epithelium (15). However, the molecular mechanisms underpinning the interplay between microbial–mucus interactions and bacterial localization in the intestinal habitat are subject to ongoing investigation.

Here, we set out to study the host receptors of an adhesin belonging to the family of multivalent adhesion molecules (MAMs)^4^ from the gut commensal *E. coli* strain HS, which was originally isolated from a fecal sample of a healthy donor (16). MAMs are abundant in Gram-negative bacteria, and in the context of pathogens they have been shown to initiate adhesion to host tissues and infection (17, 18). The *Vibrio parahemolyticus* MAM (MAM7) has been shown to bind phosphatidic acids, anionic lipids found at the host membrane, and to use the extracellular matrix protein fibronectin as a co-receptor (19). It was further shown that MAM7 binding to host cells competitively precludes other bacteria from adhering (17, 20) and that this could be utilized as a strategy to combat infection *in vivo* (21). Our recent work showed that binding of the MAM homolog from the commensal *E. coli* HS (or short, MAM^HS^) could also competitively inhibit bacterial attachment to host cells (22). Adherent invasive *E. coli* cells, which have been associated with inflammatory bowel disease, encode for a MAM that shares 99% sequence identity with MAM^HS^. However, whether it targets the same host receptors as MAM7 was unclear, and its functionality could not be deducted from the sequence, due to low sequence conservation. The commensal *E. coli* strain HS has been shown to provide colonization resistance against pathogenic *E. coli* O157:H7 in the intestine, and this has been linked to nutritional competition (23), but whether competitive adhesion to host receptors plays a role in this and what factors in *E. coli* HS contribute to host attachment is not well-understood.

Here, we show that the MAM^HS^ adhesin derived from the commensal *E. coli* strain HS binds to anionic host lipids, but in contrast to *V. parahemolyticus* MAM7 has a preference for sulfated lipids. Both MAM^HS^ and MAM7 also interact with a range of host protein receptors, including fibronectin, through a conserved 3-*O*-sulfogalactosyl moiety that is conserved between sulfoglycosylated proteins and sulfatide (3-*O*-sulfogalactosylceramide). The interaction of MAM^HS^ with sulfated mucin competitively inhibits attachment of *E. coli* to epithelial cells and enhances the retention of *E. coli* by a mucin gel. This barrier effect is significantly decreased by mucin desulfation, such as by the mucin forager *Bacteroides thetaiotaomicron*.

Our results suggest that the physical barrier effect of mucin can be modulated by specific interactions between commensal adhesins and mucin, and the interplay between mucin and microbes cohabiting the same intestinal niche as *E. coli* may modulate the localization of commensals within the gut.

## Results

### Multivalent adhesion molecules from different bacteria show conserved binding to host lipids but show preference for different anionic headgroups

**Figure 1.**
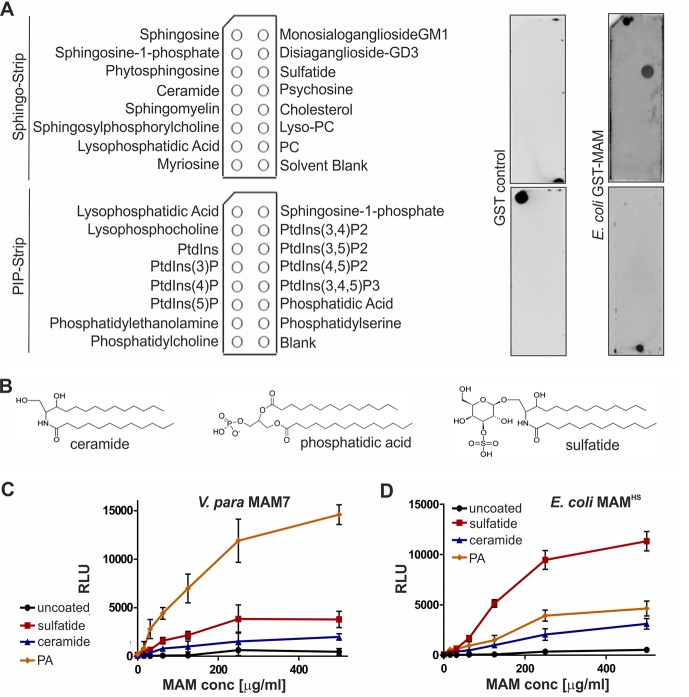
***V. parahemolyticus* MAM7 and *E. coli* MAM^HS^ show different binding preferences for host–lipid receptors.** *A,* lipid-binding profile of *E. coli* GST-MAM^HS^ and GST control was determined by lipid overlay assays. 10 μm recombinant pure proteins were incubated with Sphingo-Strips or PIP-Strips (containing 100 pmol of each lipid species), and bound proteins were detected by probing membranes with α-GST and α-mouse HRP antibodies and developed with enhanced chemiluminescence detection reagent. Pictures shown are representative of at least three independent experiments. *PC,* phosphatidylcholine; *PtdIns,* phosphatidylinositol. *B,* comparison of chemical structures of ceramide, phosphatidic acid, and sulfatide headgroups. Interactions between *V. parahemolyticus* MAM7 (*C*) or *E. coli* MAM^HS^ (*D*) and lipids were quantified using plate assays. Lipids were immobilized in wells, and bound protein was quantified by probing wells with α-GST and α-mouse HRP antibodies, developed with ECL detection reagent, and measured by relative luminescence emission (*RLU*). Results shown are means ± S.E. (*n* = 3).

We have previously shown that the MAM from the commensal *E. coli* strain HS (MAM^HS^) competitively inhibits pathogen adherence to human cells (22). However, the protein sequence of *E. coli* MAM^HS^ is quite different from the sequence of *V. parahemolyticus* MAM7, the first characterized MAM that uses the host–lipid phosphatidic acid and the extracellular matrix protein fibronectin as receptors (17, 19). To determine ligand-binding specificity, we first tested the lipid-binding properties of MAM^HS^ using lipid overlay assays. Although MAM7 from *V. parahemolyticus* bound to phosphatidic acid (17), *E. coli* MAM^HS^ bound to sulfatide (Fig. 1*A*). Like phosphatidic acid, sulfatide (3-*O*-sulfogalactosylceramide) includes a negatively charged headgroup that is necessary for binding. However, no binding was detected to ceramide, which is structurally identical to sulfatide but lacks the sulfogalactosyl moiety (Fig. 1*B*). To quantitate binding of both MAM7 and MAM^HS^ to lipids, we used a modified indirect quantitative ELISA and chemiluminescence detection. Wells of a high-bind microtiter plate were coated with sulfatide, ceramide, and phosphatidic acid or were left uncoated. GST-MAM7 or GST-MAM^HS^ was added at the indicated concentrations, and binding was quantified by incubation of plates with GST antibody followed by incubation with a secondary HRP-coupled antibody and chemiluminescence measurements. MAM7 showed strong binding to phosphatidic acid, weaker binding to sulfatide, and no significant binding to ceramide or uncoated wells (Fig. 1*C*). In contrast, MAM^HS^ bound to sulfatide with high affinity but displayed much lower binding to phosphatidic acid and ceramide (Fig. 1*D*). These data demonstrate that whereas both MAMs specifically bind to anionic host lipids, *V. parahemolyticus* MAM7 preferentially binds to phosphatidic acid, which features a glycerophosphate headgroup, whereas *E. coli* MAM^HS^ prefers sulfatide, featuring a galactosyl-sulfate moiety, as receptor.

### E. coli MAM^HS^ and V. parahemolyticus MAM7 recognize 3-O-sulfogalactosyl moieties found in host–lipid and protein receptors

**Figure 2.**
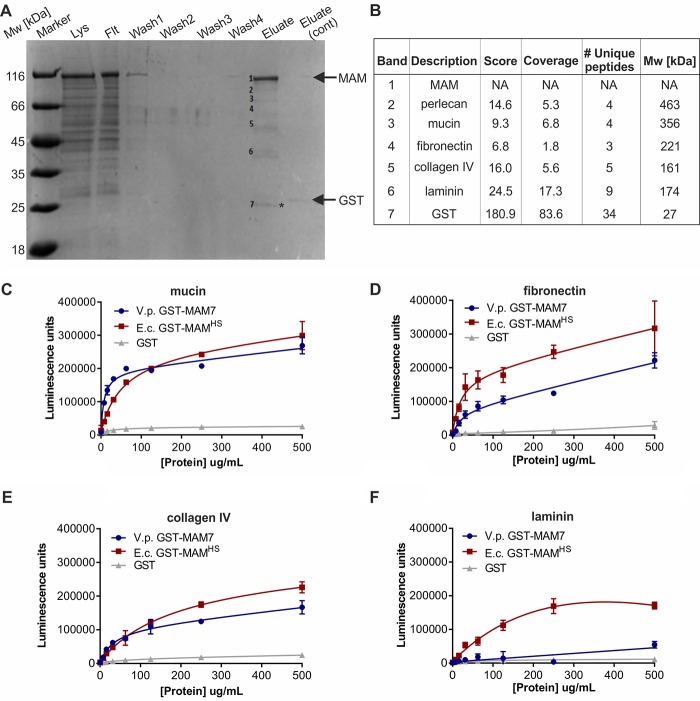
***E. coli* MAM^HS^ and *V. parahemolyticus* MAM7 interact with multiple host protein receptors.** *A,* pulldown of HeLa lysate with purified *E. coli* GST-MAM^HS^. Lysate (*Lys*), flow-through (*Flt*), wash, and eluate fractions were resolved by SDS-PAGE. A control experiment was done using GST only, and eluate from this experiment was run on the same gel (*eluate cont*.). Molecular masses of GST-MAM^HS^ and GST control are indicated by *arrows*. GST-containing band in MAM (*) and control eluate were run at slightly different heights, likely as a result of partial proteolysis. Eluted *bands 1–7* were cut out and subjected to tryptic digest and protein identification by liquid chromatography-tandem mass spectrometry. *B,* top hits for protein bands 1–7 depicted in *A*. Binding of purified GST-MAM^HS^, GST-MAM7, or GST (control) to mucin from porcine stomach type II (*C*), fibronectin (*D*), collagen IV (*E*), or laminin (*F*) was quantified using α-GST and α-mouse HRP antibodies coupled with chemiluminescence detection. Wells in *C–F* were coated with 5 μg of host protein, and 0–500 μg/ml purified GST-MAM or GST were added.

We conducted pulldown experiments with epithelial cell lysates to determine whether, like MAM7, *E. coli* MAM^HS^ would recognize host proteins as adhesion receptors. GST-MAM^HS^ was used as a bait to pull down bound proteins from HeLa cell lysate, and GST alone was used as a negative control. SDS-PAGE revealed the presence of seven specific bands, corresponding to MAM, GST, and five putative interacting proteins (Fig. 2). Bound host proteins (corresponding to bands 2–6) were identified as perlecan, mucin, fibronectin, collagen IV, and laminin as top hits. We conducted indirect ELISAs to test whether MAM^HS^ directly bound these protein receptors and to compare its binding with that of MAM7 (Fig. 2). Both MAM7 and MAM^HS^ bound to mucin, fibronectin, and collagen IV. MAM^HS^ but not MAM7 bound weakly to laminin. With the exception of mucin, which coats mucosal surfaces, all of the identified proteins are part of the extracellular matrix, and all can either be glycosulfated or, in the case of laminin, bind directly to sulfatides (24, 25). This led us to test the binding specificity of MAM^HS^ and MAM7 toward glycosulfates.

**Figure 3.**
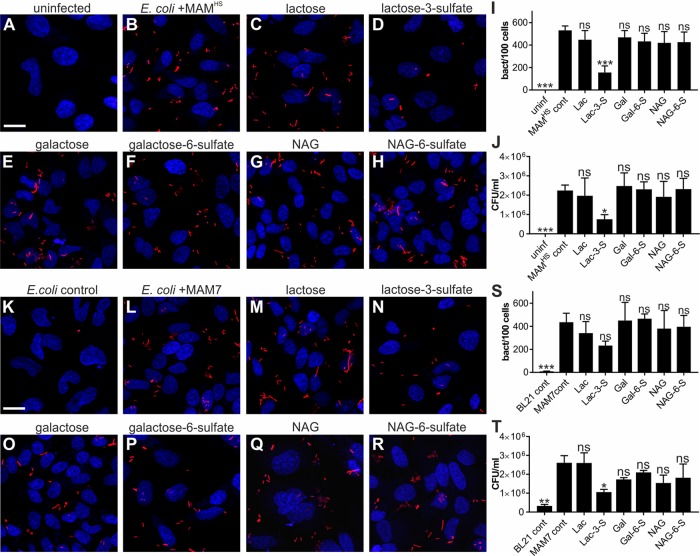
**Binding to 3-*O*-galactosyl moiety competitively inhibits attachment by *E. coli* expressing MAM^HS^ or MAM7**. Uninfected HeLa epithelial cells (*A*) and bacterial attachment of BL21-expressing mCherry and MAM^HS^ (*B*) to HeLa cells are shown. Bacterial attachment of BL21-expressing mCherry and MAM^HS^ in the presence of 200 μm lactose *(Lac, C*), lactose 3-sulfate *(Lac-3-S*, *D*), galactose (*Gal, E*), galactose 6-sulfate (*Gal-6-S*, *F*), *N*-acetylglucosamine (*NAG*, *G*), or *N*-acetylglucosamine 6-sulfate (*NAG-6-S*, *H*) is shown. Cells were fixed and stained with Hoechst (*blue*), and bacteria are visible in *red. Scale bar,* 20 μm. Bacterial attachment was quantified by image analysis (*I*), and values plotted are means ± S.E. from at least 100 cells per condition (*n* = 3). Bacterial attachment was quantified by serial dilution plating of Triton-lysed host cells (*J*), and values represent means ± S.E. (*n* = 3). Significance relative to MAM controls was determined using Student's two-tailed *t* test. *** indicates *p* ≤ 0.001; * indicates *p* ≤ 0.05; *ns* indicates *p* ≥ 0.05. Bacterial attachment of BL21-expressing mCherry (*K*) or mCherry + MAM7 (*L*) to HeLa cells is shown. Bacterial attachment of BL21-expressing mCherry and MAM7 in the presence of 200 μm Lac (*M*), Lac-3-S (*N*), Gal (*O*), Gal-6-S (*P*), NAG (*Q*), or NAG-6-S (*R*) is shown. Cells were fixed and stained with Hoechst (*blue*) and bacteria are visible in *red. Scale bar,* 20 μm. Bacterial attachment was quantified by image analysis (*S*), and values plotted are means ± S.E. from at least 100 cells per condition (*n* = 3). Bacterial attachment was quantified by serial dilution plating of Triton-lysed cells (*T*) and values represent means ± S.E. (*n* = 3). Significance relative to MAM controls was determined using Student's two-tailed *t* test. ** indicates *p* ≤ 0.01; * indicates *p* ≤ 0.05; *ns* indicates *p* ≥ 0.05.

Competitive attachment assays testing the attachment of BL21-expressing MAM^HS^ or MAM7 either in the absence or presence of small molecule inhibitors demonstrated MAM-mediated adhesion was specifically abolished by lactose 3-sulfate. The effect on binding was larger for MAM^HS^ than MAM7. In both cases, adhesion was unaffected by the presence of micromolar concentrations of lactose, galactose, galactose 6-sulfate, *N*-acetylglucosamine, or *N*-acetylglucosamine 6-sulfate (Fig. 3). Lactose 3-sulfate shares the 3-*O*-sulfogalactosyl moiety found in sulfatide. These results show that recognition is glyco-specific but also specific to the position of the sulfoglycosylation, because galactose 6-sulfate did not inhibit binding.

### Sulfation-dependent binding to mucin prevents MAM-mediated adherence to epithelial cells

Of the host protein receptors we identified, mucin is the only one exposed at the luminal side of the intestine, and it is thus likely the initial receptor encountered by bacteria entering the gastrointestinal tract and constitutes a prime target for bacterial adherence. All other identified receptors are either localized at the epithelial surface or at the basement membrane. Hence, we focused our further studies on the interaction between MAM^HS^ and mucin.

**Figure 4.**
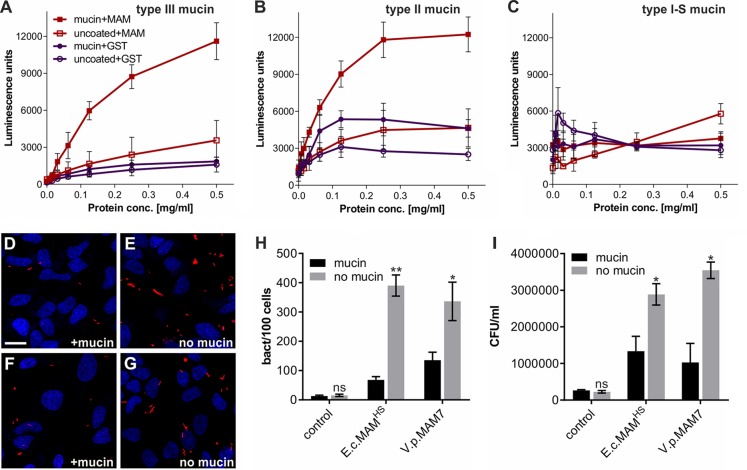
**MAM–mucin interaction competes for bacterial attachment to the host cell surface.** Binding of purified GST-MAM^HS^ or GST (control) to mucin from porcine stomach type III (*A*), mucin from porcine stomach type II (*B*), and mucin type I-S from bovine submaxillary glands (*C*) was quantified using α-GST and α-mouse HRP antibodies coupled with chemiluminescence detection. Wells contained either 5 μg of mucin or were left uncoated. GST-MAM or GST protein was added at concentrations between 0 and 0.5 mg/ml. Values represent means ± S.E. (*n* = 3). Attachment of BL21-MAM^HS^ + mCherry (*D* and *E*) or BL21-MAM7 + mCherry (*F* and *G*) to HeLa cells with (*D* and *F*) or without (*E* and *G*) prior incubation of bacteria with mucin is shown. *Blue,* Hoechst; *red,* mCherry *E. coli. Scale bar,* 20 μm. *H,* quantification of attached bacteria from experiments shown in *D–G*. Values are means ± S.E. from at least 100 cells per condition (*n* = 3). Significance of differences between mucin-treated (*black*) and -untreated (*gray*) samples was determined using Student's two-tailed *t* test. ** indicates *p* ≤ 0.01; * indicates *p* ≤ 0.05; *ns* indicates *p* ≥ 0.05. *I,* bacterial attachment was quantified by serial dilution plating of Triton X-100 lysed host cells. Values represent means ± S.E. (*n* = 3). Significance was determined using Student's two-tailed *t* test. * indicates *p* ≤ 0.05; *ns* indicates *p* ≥ 0.05.

To confirm whether MAM^HS^ and mucin directly interact, we coated microtiter plates with either mucin from porcine stomach type II, type III, or mucin type I-S from bovine submaxillary glands, and we quantitated binding of GST-MAM^HS^ using a modified indirect ELISA and chemiluminescence detection (Fig. 4). GST-MAM^HS^ strongly bound both mucin types II and III from porcine stomach (Fig. 4, *A* and *B*), whereas no binding was detected to mucin from bovine submaxillary glands (Fig. 4*C*). All three mucins contain the same Muc2 core structure but differ in their level and quality of glycosylation, with mucin type I-S carrying shorter *O-*linked glycans and being most heavily sialylated. These data suggest that MAM^HS^ specifically recognizes glycosylation patterns on mucin.

Next, we tested whether the interaction between *E. coli* expressing MAM^HS^ or MAM7 and mucin would affect the bacteria's ability to adhere to epithelial cells. Pre-incubation of MAM-expressing BL21 with mucin type II decreased bacterial attachment to HeLa cells to background levels (BL21 control without MAM), whereas the nonspecific adherence of BL21 without MAM^HS^ was not affected by incubation of bacteria with mucin (Fig. 4, *D–I*). These data demonstrate that interaction of MAMs with mucin impedes bacterial attachment to the epithelial surface, and this effect is not due to physical retention of bacteria by mucin under these experimental conditions, where mucin is soluble and at a low concentration.

**Figure 5.**
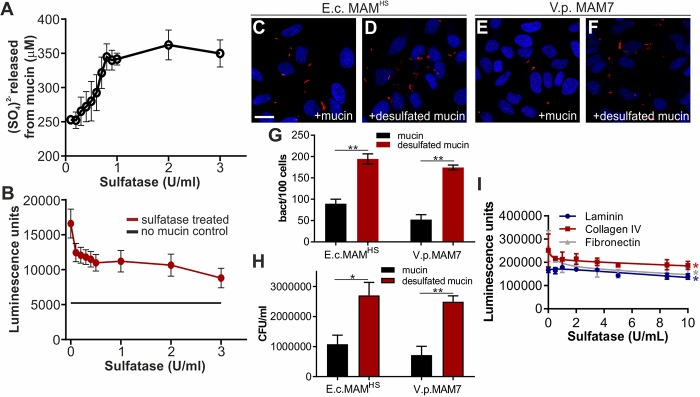
**Affinity of MAMs for host protein receptors is sulfation-dependent.** *A,* treatment with sulfatase from *H. pomatia* releases sulfate from mucin in a concentration-dependent manner. *B,* desulfation of mucin with 100 μl of sulfatase from *H. pomatia* (activities as indicated) decreases GST-MAM^HS^ binding to mucin from porcine stomach type II. Bacterial attachment of BL21-expressing mCherry and *E. coli* MAM^HS^ (*C* and *D*) or *V. parahemolyticus* MAM7 (*E* and *F*) to HeLa epithelial cells in the presence of 50 μg/ml type II mucin (*C* and *E*) or desulfated mucin (*D* and *F*) is shown. Cells were fixed and stained with Hoechst (*blue*), and bacteria are visible in *red. Scale bar,* 20 μm. Bacterial attachment was quantified by image analysis (*G*), and values plotted are means ± S.E. from at least 100 cells per condition (*n* = 3). Bacterial attachment was quantified by serial dilution plating of Triton-lysed cells (*H*), and values represent means ± S.E. (*n* = 3). Significance was determined using Student's two-tailed *t* test. * indicates *p* ≤ 0.05; ** indicates *p* ≤ 0.01. *I,* wells were coated with 5 μg of laminin (*blue*), collagen IV (*red*), or fibronectin (*gray*), which were then desulfated with 0–10 units/ml of pure sulfatase and incubated with 500 μg/ml of GST-MAM^HS^. Values represent means ± S.E. from at least three independent repeats. Significance between untreated and 10 units/ml sulfatase-treated samples was determined using Student's two-tailed *t* test. * indicates *p* ≤ 0.05.

Mucin, like the other protein receptors for MAM^HS^ and MAM7 we identified, is glycosylated, and glycosulfation constitutes one of the main glycan modifications found on gastrointestinal mucin. Sulfated residues on intestinal mucin chiefly include *N*-acetylglucosamine, *N*-acetylgalactosamine, and galactose (26–28). Because our data showed that galactose 3-sulfate is an inhibitor of bacterial attachment (Fig. 3), and a common moiety found both on sulfatides and sulfoglycosylated proteins such as mucin, we tested whether mucin sulfation was required for the interaction between MAM^HS^ and mucin. Treatment of mucin type II with purified sulfatase from *Helix pomatia* released sulfate in a concentration-dependent manner (Fig. 5*A*). Desulfated mucin was then used to test for GST-MAM^HS^ binding by a modified indirect ELISA, and desulfation decreased the binding of GST-MAM^HS^ in a dose-dependent manner (Fig. 5*B*). Desulfated mucin also lost the ability to prevent *E. coli* expressing MAM^HS^ or MAM7 from attaching to the epithelial surface, as determined both by image analysis (Fig. 5, *C–G*) and dilution plating of bacteria attached to host cells in the presence and absence of desulfated mucin (Fig. 5*H*). Treatment of fibronectin, collagen IV, and laminin with sulfatase similarly decreased their affinity for purified MAM^HS^, although residual affinity was retained even after desulfation with 10 units/ml sulfatase (Fig. 5*I*).

**Figure 6.**
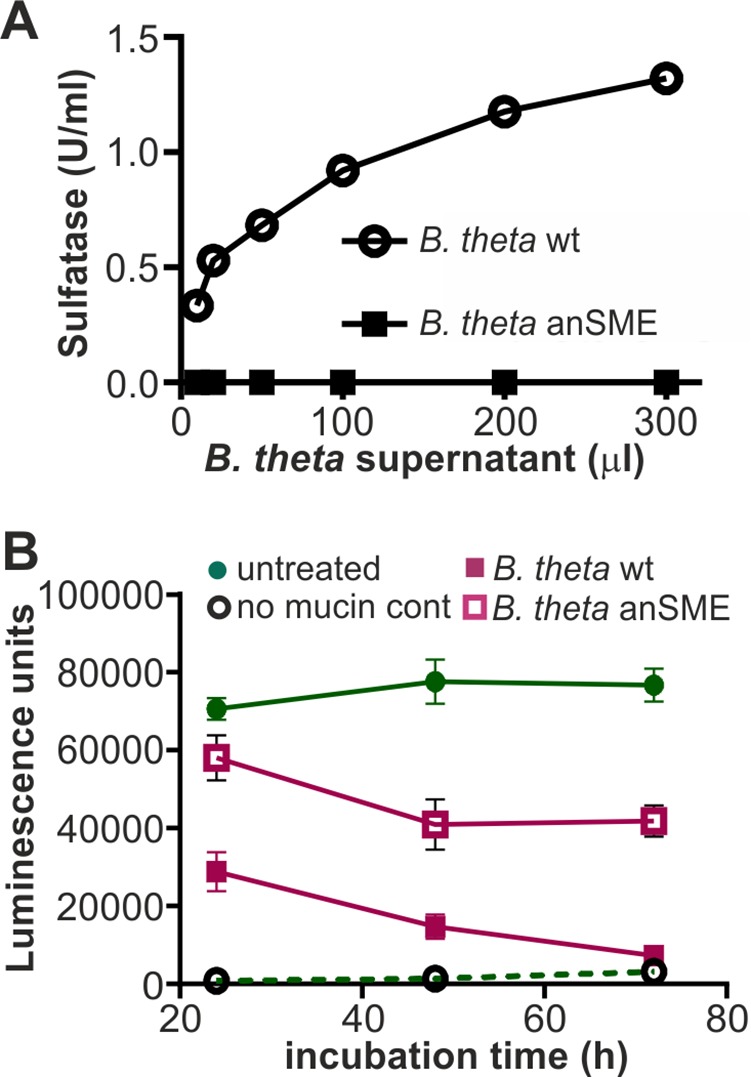
***E. coli* MAM^HS^ binding to mucin is decreased by exposure of mucin to sulfatase-secreting *B. thetaiotaomicron*.** *A,* sulfatase activity of supernatants from *B. thetaiotaomicron* WT (*circle*) and a sulfatase-deficient mutant lacking the anSME mutant (*square*) were quantified using *p-*nitrocatechol sulfate as substrate. *B,* mucin type II from porcine stomach was treated with 100 μl (1 unit/ml) of supernatant from *B. thetaiotaomicron* WT (*pink squares*) or anSME mutant (*empty squares*) for the indicated times and used in a plate assay to quantify GST-MAM^HS^ binding. As control, GST-MAM^HS^ binding to untreated mucin (*green circles*) or binding to uncoated wells (*empty circles*) was measured. All values plotted are means ± S.E. (*n* = 3).

Although *E. coli* is unable to desulfate mucin, the *Bacteroides* species, which inhabit the same gastrointestinal niche as *E. coli*, are prolific secretors of sulfatases. We therefore asked whether treatment of mucin with the secretions of the human gut symbiont *B. thetaiotaomicron* would alter MAM^HS^ binding to mucin. We determined sulfatase activity in cell-free supernatants isolated from *B. thetaiotaomicron* cultured in BHI for 48 h, as well as a sulfatase-deficient mutant (*B. thetaiotaomicron* anSME, Fig. 6*A*). We then treated type II mucin with 100 μl of *B. thetaiotaomicron* supernatant, corresponding to a sulfatase activity of 0.1 unit, which we had previously determined to be a sufficient activity to cause loss of MAM^HS^ binding to mucin (Fig. 5*B*). Treatment of mucin with supernatant from wild-type *B. thetaiotaomicron* caused a decrease of MAM^HS^ binding to mucin, which increased with increasing exposure time of mucin to the supernatant prior to incubation with MAM^HS^ and eventually led to a complete loss of binding (Fig. 6*B*). Treatment of mucin with supernatant from a sulfatase-deficient *B. thetaiotaomicron* anSME mutant caused ∼50% of the loss in MAM^HS^ binding seen with the wild-type strain after extended (72 h) incubation of supernatant with mucin and 25% of the loss after a shorter (24 h) exposure. This can be attributed to *B. thetaiotaomicron*'s ability to degrade mucin during an extended exposure. Taken together, these experiments show that the binding affinity of MAM^HS^ for mucin depends on the presence of sulfoglycan modifications on mucin, and that the exposure of mucin to sulfatase-secreting *B. thetaiotaomicron* decreases sulfation and thus the affinity of MAM^HS^ for mucin, which enables bacteria to attach to the epithelial surface.

### Mucin desulfation helps E. coli to penetrate a mucin gel

The above-described experiments suggest that the mucin-desulfating activity of sulfatase-producing bacteria cohabiting the same niche as *E. coli* may affect the ability of *E. coli* to bind to and be retained by intestinal mucus. Although these experiments conclusively demonstrate competition for *E. coli* MAM-dependent binding to mucin or the epithelial surface, they use soluble mucin and thus do not adequately reflect the situation in the gastrointestinal tract, where a continuous mucus layer, which acts as a physical barrier, precludes access of bacteria to the underlying epithelium. In such a situation, the ability of bacteria to reach the epithelium depends on both specific binding to mucin, as well as the impact of mucin modification on the structural integrity of the mucin layer. We attempted to simulate this environment by using long-term cultured mucin-producing colonic epithelial cells (HT29-MTX). However, despite our attempts to optimize culture conditions, including mucin supplementation and simulated flow, we only observed a patchy distribution of mucus and not a continuous mucus layer. Thus, we sought to simulate this more complex scenario by reconstituting a mucin gel to simulate the barrier effect of the intestinal mucus layer and test how sulfation would impact *E. coli* transmigration through this barrier.

**Figure 7.**
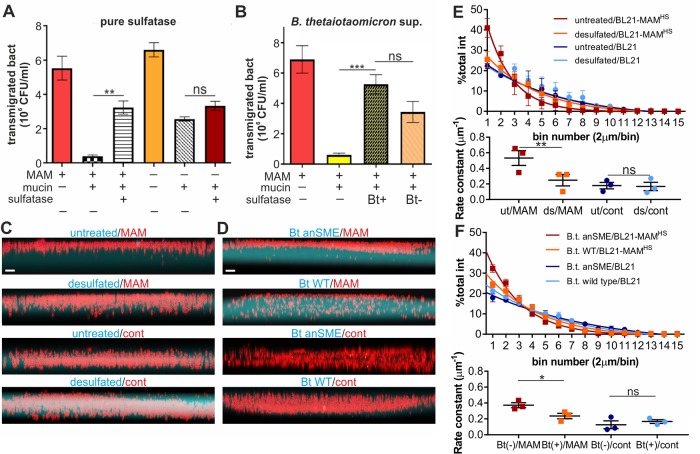
**Mucin desulfation helps *E. coli* to transmigrate a mucin gel simulating the mucus barrier.** *A,* BL21-expressing MAM^HS^ (*MAM* +) or carrying empty vector (*MAM* −) was added to the top compartment of a transwell either coated with type II mucin (*mucin* +) or left uncoated (*mucin* −). Mucin used for coating was either desulfated with sulfatase from *H. pomatia* (*sulfatase* +) or left untreated (*sulfatase* −) prior to coating transwells. Bacterial transmigration (recovery from bottom well following 2 h of incubation at 37 °C) was measured by serial dilution and plating. *B,* experiments were conducted as described in *A*, but mucin was either treated with supernatant from wild-type *B. thetaiotaomicron* (*Bt*+) or from the sulfatase-deficient *B. thetaiotaomicron* anSME mutant (*Bt*−) prior to coating transwells. Values represent means ± S.E. (*n* = 3). Significance was determined using Student's two-tailed *t* test. ***, *p* ≤ 0.001; **, *p* ≤ 0.01; *ns* indicates *p* ≥ 0.05. mCherry-BL21-expressing MAM^HS^ (*MAM*) or mCherry-BL21 carrying empty vector (*control*) were seeded on top of fluorescence-labeled, mounted gels (*cyan*) consisting of agarose and either untreated or sulfatase-treated mucin (*C*) or a gel consisting of agarose and mucin treated with *B. thetaiotaomicron* wild-type (*Bt WT*) or *B. thetaiotaomicron* anSME mutant (*Bt anSME*) (*D*). Gels were imaged using an Olympus IX83 with a Fluoview FW3000. *Scale bars,* 5 μm. Bacterial penetration of mucin gels treated with pure sulfatase (*E*) or *B. thetaiotaomicron* supernatants (*F*) was quantified from images by measuring accumulation of fluorescence in bins with 2-μm deep increments and fitting data to a one-phase decay model (*lines*). Significance was analyzed by comparing rate constants of fluorescence intensity decay over gel depth between sulfated and desulfated mucin gels. **, *p* ≤ 0.01; *, *p* ≤ 0.05; *p* ≥ 0.05 is considered not significant (*ns*).

*E. coli* rapidly migrated through uncoated transwells (pore size 3 μm), and motility was unaffected by the presence or absence of MAM^HS^ (Fig. 7*A*). Coating of transwells with a mucin gel impeded the transmigration of *E. coli* but retained *E. coli* expressing MAM^HS^ more efficiently, suggesting the mucin layer constitutes a physical barrier but its effect is enhanced through specific interactions between mucin and the MAM^HS^ adhesin. Treatment of mucin with *H. pomatia* sulfatase increased the transmigration of *E. coli* expressing MAM^HS^ to levels seen with BL21 control bacteria, whereas mucin desulfation had no significant effect on the transmigration of *E. coli* without MAM^HS^ (Fig. 7*A*). This suggests that desulfation decreases the retention of bacteria expressing the commensal adhesin MAM^HS^, whereas it has no discernible impact on the mechanical barrier effect of mucin gels. These results were recapitulated when mucin was treated with *B. thetaiotaomicron* supernatant, although in this case the permeability change is the result of both desulfation and partial mucin degradation by *B. thetaiotaomicron* (Fig. 7*B*). To better resolve the effect of mucin sulfation on bacterial penetration and decrease potential changes to mucin gel strength resulting from desulfation, we produced gels consisting of low-melting point agarose as a scaffold material, and fluorophore-labeled mucin or desulfated mucin to probe its effect on bacterial retention due to specific adhesion. Bacterial penetration into the gel following 4 h of co-incubation was visualized by confocal microscopy (Fig. 7, *C*, and *D*). Gel penetration was quantitated by measuring the fraction of bacterial (mCherry) fluorescence in 2-mm segments (bins) along the *z* axis of the gel, fitting data to a one-phase decay model and comparing rate constants (Fig. 7, *E* and *F*). Although BL21 cells expressing MAM^HS^ were efficiently retained by the gels, desulfation resulted in enhanced gel penetration, which was indistinguishable from penetration by bacteria lacking MAM^HS^. Desulfation of mucin did not affect bacterial penetration in the absence of MAM^HS^. The same results were obtained for mucin desulfated with pure sulfatase (Fig. 7, *C* and *E*) as with *B. thetaiotaomicron* supernatants (Fig. 7, *D* and *F*).

## Discussion

The protective role of the intestinal mucus layer as a barrier against microbial invasion of the underlying epithelium, and against inflammatory responses resulting from bacteria-epithelial interactions, is well-recognized (29, 30). Similarly, it has been shown that the thickness of the mucus layer and the integrity and glycosylation of its main component, the mucin protein Muc2, are key to maintaining the barrier effect (11–13). Although increased microbial penetration of the mucus layer has been linked to a loss in mucin glycosylation, the mechanisms behind these changes are not well-understood.

Here, we report the adhesin MAM^HS^ found in commensal *E. coli* as well as the *V. parahemolyticus* adhesin MAM7 interact with mucin in a sulfation-dependent manner (Fig. 5). Through a 3-*O*-sulfogalactosyl moiety shared between sulfated mucin and other host-derived proteins (Figs. 2 and 3), as well as host sulfatides (Fig. 1), MAMs can attach either to mucin or the host epithelium (Fig. 4).

Pulldown experiments with purified GST-MAM^HS^ as a bait identified five host epithelial proteins specifically interacting with the adhesin. All identified binding partners are extracellular proteins and have been reported to be heavily glycosylated and sulfoglycosylated (31–33) or to bind to sulfated ligands, such as the case of laminin that binds to sulfatides (24, 25). Four of them directly bound MAMs, albeit with varying affinities and specificity for MAM^HS^ and MAM7 (Fig. 2). As with mucin, binding to fibronectin, collagen IV, and laminin was decreased by sulfatase treatment of the receptors, but it was only partially sulfation-dependent. Perlecan was excluded from further analysis because it was not commercially available in large enough quantities. Further analysis of competitive inhibition with sugars or sulfosaccharides showed that galactose 3-sulfate is recognized by MAM and is found both in mucin and sulfatide. Although binding to other protein receptors was also decreased upon desulfation, binding was not completely lost. We therefore speculate that other receptor modifications may play a role in receptor recognition and may contribute to MAM binding. However, the chemical nature of these modifications is not as well-defined as for Muc2, hampering further detailed biochemical analysis of binding specificity. Although we show each of the identified receptors is capable of binding MAMs independently, it remains to be tested whether they are able to form multimeric complexes.

Sulfatide is an abundant host cell lipid, accounting for 4% of total membrane lipid content (34, 35). Sulfatides participate in a wide range of cellular functions, including protein trafficking, cell adhesion, and aggregation, as well as immune responses (36). Additionally, both viruses and bacteria have been found to utilize sulfatides as host receptors for adherence, including enterotoxigenic *E. coli* (37). However, the identification of 3-*O*-sulfogalactosyl as a common binding moiety promoting bacterial attachment to both host lipids and proteins, to our knowledge, has not been reported previously.

Mucin is a commonly described mediator of bacterial adherence, which is unsurprising given its abundance in the gastrointestinal tract and on other mucosal surfaces throughout the body and its role in maintaining the local microbiota. However, mucin displays a large variety of glycoepitopes and as such bacteria have evolved a diverse range of adhesin–mucin interactions. Specific interactions have been described between *Pseudomonas aeruginosa* and Lewis x, sialyl-Le(x), and sulfosialyl-Le(x) glycoepitopes and between *Helicobacter pylori* and Lewis (b) on respiratory and gastric mucins, respectively (38, 39). Targeting of human blood type A, B, or H antigens modifying intestinal mucin by *Lactobacillus* has also been reported (40, 41). *Lactobacillus reuteri* has been shown to recognize mucin in a sulfation-dependent manner, although the exact functional epitope remains elusive (42). The interactions between microbes and mucin oligosaccharides have been shown to facilitate bacterial clearance and to inhibit colonization of epithelial cells (43).

Sulfation of colonic mucins has been shown to play a protective role in animal models of colitis (26), and reduced sulfation has been associated with impaired barrier function and with infectious disease (44, 45). Our data show that the sulfation status of mucin also impacts specific bacterial adherence to and thus retention of bacteria by the mucin gel. Sulfatases produced by anaerobes colonizing the intestinal tract, such as the commensal *B. thetaiotaomicron*, are essential for mucosal foraging and have a direct impact on the chemical composition and architecture of the mucus layer (46, 47). Enhanced sulfatase activity by the intestinal microbiota has been detected in fecal samples from patients suffering ulcerative colitis (48).

Our data suggest that the level of sulfatase production, and thus mucin desulfation, can act to modulate the adherence of commensals to mucin and may directly impact their retention by the mucus barrier. A limitation in predicting *in vivo* modulation of the mucus barrier effect from our *in vitro* retention assays is that the physiological mucus layer has components in addition to mucin, which contribute to the barrier's structure and function. Additionally, mucus viscosity is not uniform, because the barrier consists of two layers: a loose outer layer and a denser, cell-adherent layer that is largely free of microbes in the healthy intestine. This complexity cannot be fully reproduced by our transmigration and penetration assays. The differences we observe regarding retention in the transmigration and penetration assays do suggest the mucin desulfation activity of sulfatase producers such as *B. thetaiotaomicron* have to be viewed in the context of their other mucin-degrading activities, which are interdependent and affect not only specific adhesion but other physicochemical properties, including gel strength. Although the experiments herein focused on the biochemical determinants of MAM^HS^–receptor interactions, future work will aim to directly assess the impact of sulfatase production on commensal adherence in a physiologically relevant model. Such experiments will determine whether the observed changes in adherence in response to desulfation described herein may have an impact on bacterial localization and mucosal inflammation *in vivo*.

## Experimental procedures

### Bacterial strains and growth conditions

Bacterial strains used in this study were *E. coli* HS (accession no. ABV06236.1; GI:157066981) and *B. thetaiotaomicron* (wild-type strain VPI-5482) and a derivative strain lacking the anaerobic sulfatase-maturating enzyme (anSME), ΔBT0238, that is unable to produce functional sulfatases (46). *E. coli* BL21-expressing MAM^HS^ or *E. coli* BL21 carrying empty pBAD (control) were grown in LB medium at 37 °C. MAM expression was induced in mid-log phase cultures by adding 0.05% arabinose and growing cells for a further 3 h. *B. thetaiotaomicron* was grown in BHI medium at 37 °C in an anaerobic jar.

### Protein expression and purification

10 ml of LB containing 100 μg/ml ampicillin was inoculated with a colony of *E. coli* BL21 containing the recombinant plasmid (pGEX-4T3-MAM7ΔTM or MAM^HS^ΔTM) for production of GST-MAM7 or GST-MAM^HS^ protein, or *E. coli* BL21 containing pGEX-4T3 plasmid for production of GST protein, and incubated at 37 °C for 16 h with shaking. Protein expression and purification were then carried out as described previously (17, 22).

### Lipid overlay assays

PIP-Strips and Sphingo-Strips (Echelon Biosciences) were used to test the lipid-binding properties of GST and GST-MAM^HS^. Each membrane was spotted with 100 pmol of each lipid species. Membranes were blocked with blocking buffer (0.1% v/v Tween 20, 5% skim milk in PBS) for 1 h at room temperature with gentle shaking. 10 μm protein was added and incubated for 1 h with shaking. Strips were washed with washing buffer (0.1% v/v Tween 20 in PBS) three times for 10 min each. GST antibody (1:1000 in blocking buffer) was applied and incubated for 1 h at room temperature with shaking. Membranes were washed with washing buffer for 10 min; this step was repeated three times. The secondary-anti mouse IgG-HRP antibody (1:5000 in blocking buffer) was added and incubated for 1 h at room temperature with shaking. Strips were then washed with washing buffer three times for 10 min each and developed using Clarity ECL^TM^ Western substrate.

### Protein–protein and protein–lipid interaction plate assays

Protein binding to immobilized lipids or protein receptors was measured by a modified version of an indirect quantitative ELISA as follows. Mucin from porcine stomach type II, mucin from porcine stomach type III, and mucin from bovine submaxillary glands (Sigma), as well as fibronectin from human plasma, collagen IV, and laminin from human placenta, were used to study the binding to MAM^HS^ and MAM7. Host proteins were dissolved in PBS at 4 °C for 20 h with gentle shaking. For immobilization, 100 μl of host proteins at a concentration of 50 μg/ml was added to 96-well high-binding microtiter plates; plates were sealed and incubated at room temperature for 20 h. Plates were washed once with PBS, and 150 μl/well of blocking buffer (1% BSA in PBS) was added, and plates were sealed and incubated at room temperature for 1 h with gentle shaking. Then the wells were washed three times with PBS + 0.05% Tween 20. Next, 100 μl/well of serial dilutions of MAM^HS^ in PBS (concentrations as indicated in figures) were added, and plates were sealed and incubated for 2 h at room temperature with gentle shaking. The wells were washed three times with washing buffer. 100 μl/well of GST antibody (1:1000 in blocking buffer) was added; plates were sealed and incubated for 1 h at room temperature with gentle shaking. Wells were washed three times with washing buffer, and 100 μl/well secondary anti-mouse IgG-HRP antibody (1:5000 in blocking buffer) was added; plates were sealed and incubated for 1 h at room temperature with gentle shaking. The wells were washed three times, and 100 μl/well Clarity ECL^TM^ Western substrate was added for detection. Chemiluminescence was visualized on a Bio-Rad Imaging system and quantified in a FLUOStar Omega plate reader. For experiments with desulfated host proteins, mucin, fibronectin, collagen IV, or laminin was desulfated with 100 μl of sulfatase from *H. pomatia* (Sigma), which was added at concentrations ranging from 0.1 to 10 units/ml in 40 mm sodium acetate buffer, pH 5, and incubated at room temperature for 24 h with gentle shaking. Following desulfation, proteins were washed three times with washing buffer and used for plate assays as described above. Alternatively, mucin was desulfated by incubation with supernatants of *B. thetaiotaomicron* WT and *B. thetaiotaomicron* anSME mutant cultures grown to *A* 0.6 under anaerobic conditions at 37 °C for the indicated time points. Following desulfation, mucin was washed, blocked, and used for plate assays as described above.

To test binding of purified MAM7 and MAM^HS^ to lipids, 3-*O*-sulfo-d-galactosyl-β-1–1′-*N*-lignoceroyl-d-erythrosphingosine, *N*-lignoceroyl-d-erythrosphingosine, and 1,2-dioleoyl-*sn*-glycero-3-phosphate (Avanti Polar Lipids) were dissolved in chloroform/methanol/water at a ratio of 2:1:0.1. 50 μl of lipids at a concentration of 200 μg/ml were immobilized in 96-well glass microtiter plates and left at room temperature for 20 h for solvent evaporation. Control wells contained only solvents but no lipid. 150 μl/well of blocking buffer (1% BSA in PBS) was added, sealed, and incubated at room temperature for 1 h. Wells were washed three times, and protein binding was probed as described above for protein–protein interaction plate assays.

### Measurement of sulfate release from mucin

Mucins at a concentration of 50 μg/ml were desulfated as described above. In triplicate, 100 μl of supernatant from each reaction was transferred into a 96-well microtiter plate, and 100 μl of a 20% barium chloride solution was added to each well. The absorbance (*A*_600_) was measured and converted into sulfate concentration using a standard curve of Na_2_SO_4_.

### Measurement of sulfatase activity

*B. thetaiotaomicron* WT and *B. thetaiotaomicron* anSME mutant were cultured in BHI containing 2.5 mg/ml mucin from porcine stomach type II and incubated anaerobically at 37 °C for 48 h. The supernatant was collected after centrifuging cultures at 13,000 × *g* for 5 min. Serial dilutions from the supernatant were prepared using 0.2% NaCl. 100 μl of supernatant were incubated with 500 μl of 200 mm sodium acetate buffer and 400 μl of 6.25 mm *p-*nitrocatechol sulfate solution at 37 °C for 30 min. Then 5 ml of 1 n NaOH was added to stop the reaction. The absorbance at 515 nm was detected using a Jenway 6300 UV-visible spectrophotometer. Sulfatase from *H. pomatia* (Sigma) was used to prepare the standard curve.

### Protein pulldown experiments and protein identification

HeLa cells (ATCC clone CCL-2) in confluent growth were harvested using a cell scraper and centrifuged at 1000 × *g*, 22 °C for 5 min. The pellet was washed in ice-cold buffer (20 mm Hepes-KOH, pH 7.3, 110 mm KAc, 2 mm MgAc, 1 mm EGTA, 2 mm DTT), and 1 ml of lysis buffer (10 mm Hepes-KOH, pH 7.3, 10 mm KAc, 2 mm MgAc, 2 mm DTT, protease mixture (Roche Applied Science)) was added and left on ice for 10 min. Cells lysate was centrifuged at 12,000 × *g* for 12 min. 200 μg of GST-MAM^HS^ or GST (control) was added to the cell lysate and incubated for 5 min at room temperature. Dithiobis(succinimidyl propionate) cross-linker in DMSO was added to give a final concentration of 100 μg/ml and incubated at room temperature for 30 min. Tris-HCl, pH 8.0, was added to 10 mm to quench the reaction, and the solution was added to glutathione-Sepharose beads, incubated for 2 h at room temperature and then 20 h at 4 °C. The suspension was centrifuged, and the pellet was washed four times with washing buffer (20 mm Hepes-KOH, pH 7.3, 110 mm KAc, 2 mm MgAc, 1 mm EGTA, 2 mm DTT, 0.1% Tween 20, 150–500 mm NaCl). Proteins were eluted into SDS sample loading buffer, boiled, and resolved by SDS-PAGE. Gels were Coomassie-stained, and bands were cut out and sent for protein tryptic digest and identification of peptides by liquid chromatography tandem-mass spectrometry on a Thermo Orbitrap Elite system coupled to a Dionex nanoLC (University of Birmingham Advanced Mass Spectrometry Core).

### Bacterial attachment assays

HeLa cells at a concentration of 1.5 × 10^5^ cells/well were seeded on coverslips 3 days before the experiments. Colorless DMEM containing *E. coli* BL21-expressing MAM^HS^ or MAM7 at an multiplicity of infection of 100 was added to cells. For competition experiments, infection medium also contained 50 μg/ml mucin or desulfated mucin, or lactose 3-sulfate, lactose, *N*-acetylglucosamine 6-sulfate, *N*-acetylglucosamine, galactose 6-sulfate, or galactose at a concentration of 200 μm and was pre-incubated with bacteria for 1 h prior to addition to cultured cells. Following bacterial adhesion, DMEM was removed, and cells were washed three times with PBS to remove non-adherent bacterial cells, and 0.5% Triton X-100 in PBS was added to lyse mammalian cells. Serial dilutions were prepared and cultured on LB agar at 37 °C for 24 h to determine colony-forming units. To visualize bacterial attachment to host cells, bacteria were co-transformed with pDP151 (mCherry) to give constitutive red fluorescence. DMEM was removed following the adhesion experiment, and cells were washed three times with sterile PBS. For fixation, 3.7% paraformaldehyde in PBS was added for 15 min at room temperature. Cells were washed, permeabilized with 0.1% Triton X-100 in PBS for 5 min, and stained with Hoechst and Alexa488-phalloidin for 10 min. Coverslips were mounted using Prolong Antifade mountant and imaged on a Olympus IX83 fitted with a FV3000 confocal system using a UPLFLN U Plan, ×20 objective (N.A.0.5). Images were acquired using Olympus CellSens software and processed using ImageJ and Corel Draw X8 Graphics Suite.

### Mucin transmigration assays

Transwell filters (24-well thincert, 3.0-μm pore diameter, Greiner Bio-One) were coated with 50 μl of 10 mg/ml mucin from porcine stomach type II at 4 °C for 20 h, then placed onto 24-well plates containing 600 μl of DMEM without phenol red. 100 μl of 10^6^ cfu/ml *E. coli* was added to the top well and incubated at 37 °C for 2 h. Bacterial concentrations in the bottom well were enumerated by dilution plating on LB agar following incubation at 37 °C for 20 h. To study the effect of sulfatase and *B. thetaiotaomicron* on bacterial transmigration, *B. thetaiotaomicron* WT and *B. thetaiotaomicron* anSME mutant were grown anaerobically in BHI at 37 °C to an *A*_600_ of 0.6. Mucin was treated with 100 μl of culture supernatant from *B. thetaiotaomicron* WT or *B. thetaiotaomicron* anSME mutant at 37 °C for 24 h. Alternatively, mucin was treated with *H. pomatia* sulfatase as described above, prior to use in transwell assays.

### Gel penetration assays

Solutions (1 mg/ml in PBS) of untreated or desulfated (10 units/ml sulfatase, 37 °C for 2 h) mucin were prepared, and Alexa Fluor 488 succinimidyl ester was added at a 5-fold molar excess. The reaction was maintained at room temperature for 1 h and quenched by adding 100 mm Tris, and excess fluorophore was removed by buffer exchange into PBS using centrifugal filters with a 30-kDa molecular mass cutoff. 1% low-melt agarose in PBS was mixed with 50% (v/v) labeled mucin, and gels were cast into glass-bottom plates. Gels were incubated with 50 μl of *A* 1.0 cultures of BL21 mCherry expressing MAM^HS^ or carrying vector control for 4 h at 37 °C. Supernatant was removed, and gels were fixed by adding 3.7% paraformaldehyde in PBS for 15 min at room temperature. Gels were imaged using an Olympus IX83 fitted with FV3000 confocal system using a UPLFLN U Plan, ×20 objective (N.A.0.5). Red fluorescence intensities were quantified in bins with 2-μm deep increments across the *z* axis, and binned intensities were expressed as fractions of total fluorescence intensity across the entire z-stack. To determine differences between penetration profiles, data were fitted to a one-phase decay model, and rate constants were compared using a one-way analysis of variance and Dunnett's correction for multiple comparisons.

## Author contributions

F. A., D. P. V, D. H. S., and A. M. K. did experiments and analyzed data. F. A. and A. M. K drafted the manuscript. All authors read, edited, and agreed to the final version of the manuscript.

## Footnotes

4 The abbreviations used are:

MAM: multivalent adhesion molecule
MAM^HS^: MAM from the gut commensal *E. coli* HS
anSME: anaerobic sulfatase-maturating enzyme
BHI: brain heart infusion.
